# Periodic cycles of pneumococcal serotypes carried by children before and after 7-valent pneumococcal conjugate vaccine

**DOI:** 10.1371/journal.pone.0176723

**Published:** 2017-04-28

**Authors:** Ana Cristina Paulo, Raquel Sá-Leão

**Affiliations:** 1 Laboratory of Molecular Microbiology of Human Pathogens, Instituto de Tecnologia Quimíca e Biológica António Xavier, Universidade Nova de Lisboa, Oeiras, Portugal; 2 Departamento de Biologia Vegetal, Faculdade de Ciências, Universidade de Lisboa, Lisboa, Portugal; University of Waterloo, CANADA

## Abstract

**Background:**

Periodic cycles in the serotype-specific incidence of invasive pneumococcal disease have been described but less is known in carriage.

**Methods:**

We analyzed serotype carriage prevalence among children 0–6 years old over a 15-year period that included pre-PCV7 data and a decade of PCV7 use. Mixed generalized additive models were used to study periodic cycles and how PCV7 impacted on them.

**Results:**

Pneumococcal carriage data of 7,463 children were analyzed. Periodic cycles ranging from 3 to 6 years were observed for PCV7-serotypes (VT) 14, 19F and 23F and for non-PCV7 types (NVT) 3, 6A, 6C, 11A, and NT. An indirect impact of PCV7 on periodic cycles of NVT was observed and could be translated in three ways: (i) a higher amplitude in the PCV7 period (serotypes 3 and 11A), (ii) sustained increase in the prevalence of carriage (serotypes 6C, 19A and NT) and (iii) an increase in the inter-epidemic period (serotypes 3, 6A and NT). An increase in the child’s mean age of carriage of VTs 6B, 19F and 23F was observed. Serotypes 3, 6C, 11A and 15A became more frequent in ages previously associated with carriage of VTs.

**Conclusions:**

Periodic cycles among serotypes frequently carried exist and can be modeled. These cycles can be perturbed upon introduction of PCVs and can lead to shifts in the mean age of carriage. Cyclic re-emergence of VTs can occur in settings with non-universal vaccine use. These results should be taken into account when interpreting surveillance data on pneumococcal carriage.

## Introduction

*Streptococcus pneumoniae* (pneumococcus) is an obligate colonizer of humans and is commonly isolated from the nasopharynx of children younger than 6 years old [1]. Before the availability of conjugate vaccines, pneumococci were responsible for an estimated 14.5 million episodes of serious pneumococcal disease worldwide, and for 11% of all the deaths among children [2]. In countries where seven-valent pneumococcal conjugate vaccine (PCV7—targeting serotypes 4, 6B, 9V, 18C, 19F and 23F of the 97 described to date) was introduced, a sustained decrease in the number of cases of invasive pneumococcal disease (IPD) was observed [3]. Among vaccinated children pneumococcal carriage has remained stable but extensive serotype replacement was found to occur [4,5]. In 2010, a 13-valent conjugate vaccine (PCV13), targeting PCV7 types and six additional serotypes (1, 3, 5, 6A, 7F and 19A) was introduced and further declines in the incidence of IPD and serotype replacement in carriage have been documented [6–9].

Competition between serotypes has been cited as the most likely hypothesis to explain the emergence or expansion of serotypes, after decline of PCV7-serotypes, known as serotype replacement [5,10]. Nonetheless, temporal oscillations in carriage of pneumococcal serotypes can be an additional factor to explain sudden changes in prevalence of non-vaccine as well as of vaccine-serotypes. Furthermore, since periodic cycles were observed for invasive pneumococcal disease [11–14] we hypothesized that the same pattern may be occurring in carriage.

In this paper we investigated whether periodic cycles of carriage of pneumococcal serotypes exist among children and how PCV7 impacted on those cycles. We used a dataset spanning from the pre-vaccine era until the end of the PCV7 era (1996–2010). We show that the carriage of pneumococcal serotypes exhibits periodic cycles and these can explain counter-intuitive changes in prevalence of specific serotypes following PCV7 introduction. The results clearly demonstrate that to understand serotype replacement due to use of PCVs, not only PCVs, but also periodic serotype cycles, have to be taken into consideration.

## Methods

### Study setting

In Portugal, PCV7 became available in June 2001 and PCV13 in January 2010. The Portuguese Pediatric Society recommended vaccination of young children with PCV [15] but until June 2015, PCV was not reimbursed by the state nor was included in the National Immunization Plan. Nonetheless, national estimates indicate that PCV7 usage reached 56% in 2003 and 79% in 2007. In 2009, PCV7 coverage estimate was 62% (data from IMS and INE/National Statistics Institute).

Between 1996 and 2010 children up to six years old attending day-care centers in the urban region of Lisbon/Oeiras, Portugal were enrolled in the study as described elsewhere [16]. Briefly, a trained nurse obtained one nasopharyngeal sample, for each child. Sampling took place in the first three months of each year, between 1996 and 2010 with the exception of 2000, 2004, 2005 and 2008 when no sampling occurred. Data on demographic and clinical information were obtained from the child's guardians. Approval for this study was obtained from the Ministry of Education and the day-care centers directors. Signed informed consent was obtained from the parents or child's guardians. Children records were de-identified and analysed anonymously and the strains, not human subjects, were studied. Isolation and serotyping of pneumococcus was previously described [16]. Briefly, pneumococci were identified based on colony morphology, occurrence of α-hemolysis on blood agar plates and optochin susceptibility. Capsular type was determined by the Quellung reaction or by sequential multiplex PCR using primers previously described ([17] and [18]. Suspected non-capsulated pneumococci (NT, non-typeable) were identified using a multiplex PCR-based strategy previously described [19].

### Trends of vaccine coverage and pneumococcal carriage

PCV7 coverage per year in the study population was estimated as the ratio between the number of PCV7-vaccinated children and the total number of children sampled. The chi-squared test for trend in proportions was used to analyze the trends on vaccine coverage and on pneumococcal carriage. A Student’s t-test was used to compare two mean values.

### Periodic cycles of specific serotypes

Periodic cycles were investigated for all serotypes with a prevalence of at least 2.0% in each vaccine period (pre-, low- and high-, as defined below).

A mixed generalized additive model (GAM) [20] with a logit link was used to investigate whether periodic cycles in the prevalence of each serotype occurred. The initial model included as independent variables, the year of sampling, vaccine coverage, children’s age and day-care center unit. However, since both the year of sampling and vaccine coverage were strongly correlated, this led to problems of singularity. To overcome the problem vaccine coverage was grouped into three periods (pre-vaccine (1996–2001), low-vaccine coverage (2002–2003) and high-vaccine coverage (2006–2010)). Day-care centers were included in the model as a random effect to account for possible clusters on pneumococcal serotype transmission. A cyclic version of a P-spline was used for the variable “year” and a random effect smooth spline was used for the variable “day-care center”.

The model was fitted to the carriage data. For each serotype a model was fitted and used to explore the association between carriage of specific serotypes and PCV7 coverage. The strength of the associations was given in adjusted odds ratio (OR_adj_) and the corresponding confidence interval at 95% (CI 95%). An OR was considered statistically significant when the confidence interval was below 1, indicating a protective factor for carriage, or above 1, indicating a risk factor for carriage. The goodness of fit of the model was assessed by the Hosmer-Lemeshow test and the Akaike Criterium [20].

The period of time including the pre- and low-vaccine period were used to uncover the occurrence of periodic peaks before extensive PCV7 use. Multi-taper spectral analysis and the harmonic F-test were used to test the significance of peaks in serotype carriage in that time-period [21]. Spectral analysis was applied to the detrended data, calculated by subtracting the fitted regression line from the original data. A second generalized linear model, in which age was the dependent variable and the vaccine period the independent variable, was used to explore the effect of vaccine coverage on children’s age at carriage.

The statistical analyses were done using R version 3.1.1 [22].

## Results

### Population characteristics, pneumococcal carriage and serotypes

Between 1996 and 2010, 7,463 nasopharyngeal samples were obtained from children attending 39 day-care centers. PCV7 coverage in the study population increased significantly from 0% in the pre-vaccine period, to 15.9% in the low-vaccine period, and 70.3% in the high vaccine period (*P*≤0.001, Table 1).

**Table 1:** Characteristics of the population under study stratified by PCV7 coverage.

Period	No. children	Age (yrs), Mean ± SD^a^	PCV7 coverage, % (95% CI)	Carriage prevalence, % (95% CI)	Most prevalent serotypes	
					(~50% among total isolates; 95%CI)	PCV7 type
Pre-vaccine (1996–2001)	3757	3.46±1.56	-	56.7 (55.1–58.2)	6B (14.0; 12.6–15.6)	Yes
					23F (12.1; 10.7–13.5)	Yes
					19F (9.7; 8.5–11.0)	Yes
					6A (8.0; 6.9–9.3)	No
					14 (7.8; 6.7–9.0)	Yes
Low-vaccine (2002–2003)	1598	3.50±1.52	15.9 (14.2–17.8)	69.5 (67.2–71.7)	19F (12.9; 11.0–14.9)	Yes
					6B (8.7; 7.2–10.5)	Yes
					23F (8.1; 6.6–9.8)	Yes
					6A (6.6; 5.2–8.2)	No
					3 (6.1; 4.8–7.7)	No
					11A (6.0; 4.8–7.6)	No
					14 (5.7; 4.5–7.3)	Yes
High-vaccine (2006–2010)	2108	3.38±1.52	70.3 (68.3–72.2)	63.6 (61.5–65.6)	19A (12.5; 10.8–14.3)	No
					6C (8.8; 7.5–10.5)	No
					3 (6.6; 5.4–8.1)	No
					6A (6.1; 4.9–7.5)	No
					NT (5.6; 4.5–7.0)	No
					19F (5.4; 4.3–6.7)	Yes
					15A (5.2; 4.1–6.5)	No

^a^ SD for standard deviation.

Overall, 63 capsular types and non-typeable isolates were identified. Serotype distribution was detailed elsewhere [16]. The prevalence of pneumococcal carriers and the most abundant serotypes at each period are indicated in Table 1.

### Periodic cycles on the carriage prevalence of PCV7 serotypes

PCV7 serotypes 4, 9V and 18C were carried infrequently (serotype 4 was not found, serotypes 9V and 18C were carried by less than 0.5% of the children) and were not analyzed. The prevalence of serotypes 6B, 14, 19F and 23F was found to be associated with the vaccine period after adjusting for the child’s age, calendar year and day-care center (Table 2). Specifically, the high-vaccine period was associated with a significant decrease in the prevalence of serotypes 6B, 14 and 23F.

**Table 2:** Effect of PCV7 coverage on carriage of PCV7 and non-PCV7 serotypes.

Serotype	Pre-vaccine		Low-vaccine		High-vaccine	
	Carriage (%)	OR_adj_ (CI 95%)	Carriage (%)	OR_adj_ (CI 95%)	Carriage (%)	OR_adj_ (CI 95%)
6B	14.0	Ref.	8.7	0.62 (0.38–1.01)	0.4	**0.03 (0.01–0.08)**^**a**^
14	7.8	Ref.	5.7	0.79 (0.32–1.93)	2.6	**0.20 (0.06–0.68)**^**a**^
19F	9.7	Ref.	12.9	**1.64 (1.06–2.54)**^**a**^	5.4	1.00 (0.58–1.72)
23F	12.1	Ref.	8.1	0.58 (0.30–1.13)	1.9	**0.09 (0.03–0.23)**^**a**^
3	4.7	Ref.	6.1	0.15 (0.03–1.75)	6.6	1.69 (0.71–3.99)
6A	8.0	Ref.	6.6	**0.22 (0.11–0.44)**^**a**^	6.1	0.10 (0.04–0.27)
19A	4.1	Ref.	5.7	**1.94 (1.21–3.12)**^**a**^	12.5	**5.10 (3.05–8.53)**^**a**^
6C	1.0	Ref.	2.1	2.82 (0.37–21.62)	8.8	**7.36 (2.19–24.76)**^**a**^
11A	3.4	Ref.	6.0	2.76 (0.66–11.61)	3.4	1.40 (0.41–4.72)
15A	1.2	Ref.	1.6	5.58 (0.60–51.80)	5.2	2.70 (0.69–10.65)
15B/C	2.7	Ref.	4.6	**1.58 (1.00–2.51)**^**a**^	4.7	1.44 (0.89–2.34)
NT	2.4	Ref.	5.1	**6.82 (2.31–20.13)**^**a**^	5.6	**5.10 (1.71–15.22)**^**a**^

The odds ratio (OR) was adjusted for the child’s age, fluctuations over the years, and day-care center in a mixed generalized additive model with a logit function. The day-care center was introduced in the model as a random variable.

^a^statistically significant

**Fig 1:**
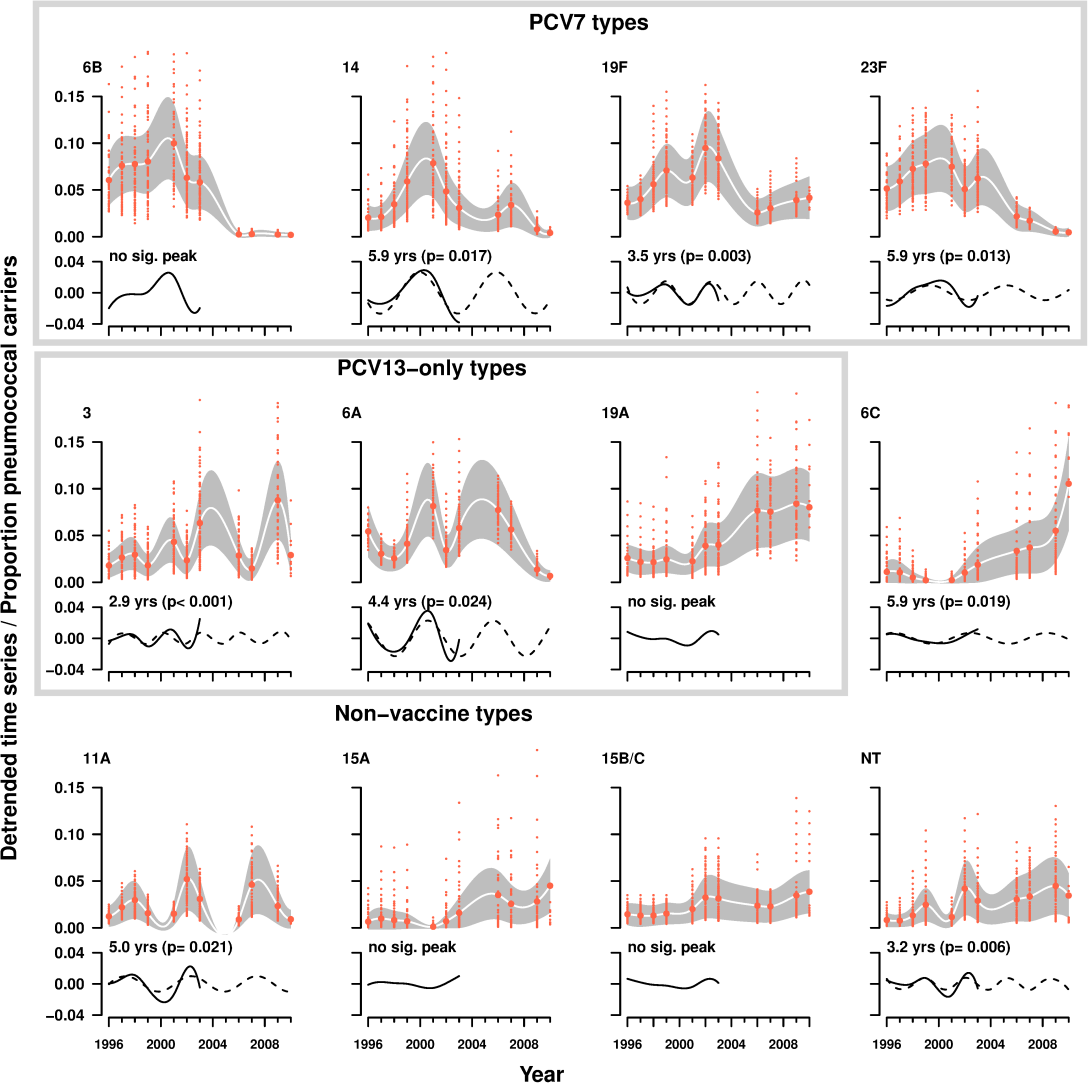
Proportion of carriers according to pneumococcal serotype as a function of time. Panels show the fit (white line) of a mixed generalized additive model to the proportion of carriers of specific serotypes per year, and the corresponding harmonic period estimated from the detrended time series from 1996 to 2003. For the detection of significant harmonic peaks we used 7 tappers and a time-bandwidth of 0.56. Large red dots, indicate mean proportion of carriers by year. Small red dots indicate the proportion of carriers by day-care center and child age. The gray shaded area represents the confidence interval at 95% estimated from the fit of the model. In the detrended time-series the solid line represents the expected cycle whereas the dashed lines represents the cycle observed for the pre- and low-PCV7 periods.”

Analysis of the pre- and low-vaccine periods showed periodic cycles for serotypes 14, 19F and 23F with inter-epidemic periods of 5.9, 3.5 and 5.9 years, respectively (Fig 1). No significant periodic cycles were observed for serotype 6B. Of note, serotype 14 peaked in 2006–2007 (high-vaccine period) following the predicted cyclic pattern for this serotype albeit with a lower prevalence when compared to the pre-vaccine period (Fig 1).

Comparison of the mean age of carriage of PCV7 serotypes between the high-vaccine and pre-vaccine periods indicated that it increased significantly for serotypes 6B (from 2.8 to 4.2 yrs), 19F (from 3.0 to 3.7 yrs) and 23F (from 2.9 to 3.9 yrs) (Table 3). Overall age distribution (which was not different over time) and the proportion of carriage of specific serotypes within different age groups over time are shown in S1 Fig.

**Table 3:** Mean age of carriage of specific serotypes in the pre-vaccine and high-vaccine period.

	Mean age ± standard deviation (yrs)		
Serotype	Pre-vaccine	High-vaccine	P
6B	2.8 ± 1.5	4.2 ± 1.3	0.006
14	2.6 ± 1.5	3.5 ± 1.6	0.102
19F	3.0 ± 1.7	3.7 ± 1.5	0.004
23F	2.9 ± 1.7	3.9 ± 1.1	<0.001
3	4.4 ± 1.0	3.9 ± 1.0	0.005
6A	3.3 ± 1.6	3.5 ± 1.4	0.093
19A	2.9 ± 1.9	2.9 ± 1.6	0.918
6C	3.3 ± 1.7	2.8 ± 1.5	0.031
11A	4.1 ± 1.3	3.4 ± 1.8	0.012
15A	3.0 ± 1.5	2.6 ± 1.5	0.036
15B/C	3.7 ± 1.4	3.6 ± 1.6	0.491
NT	2.8 ± 1.6	3.3 ± 1.4	0.301

The trend in the mean age of carriage, for each serotype, was estimated from a generalized linear model in which age was a dependent variable and the vaccine period the independent variable.

### Periodic cycles on the carriage prevalence of PCV13 serotypes not included in PCV7

Serotypes 1, 5, and 7F were carried infrequently (less than 2% of children) and were not analyzed. The prevalence of serotypes 6A and 19A was found to be associated with the vaccine period after adjusting for the child’s age, calendar year and day-care center (Table 2). Specifically, the high-vaccine period was associated with a significant increase in the prevalence of serotype 19A. No significant change was found in the prevalence of serotype 3 between vaccine periods.

Analysis of the pre- and low-vaccine periods showed periodic cycles for serotypes 3 and 6A with inter-epidemic periods of 2.9 and 4.4 years, respectively (Fig 1). No significant cycles were observed for serotype 19A. Although PCV7 did not directly target these serotypes, during the high-vaccine period, carriage of serotype 6A, peaked one year earlier (2006) than expected and decreased significantly in 2009–2010. Also, serotype 3 peaked in 2009, one year later than the year predicted by the model and with a higher prevalence.

Comparison of the mean age of carriage of PCV13 serotypes not targeted by PCV7 between the high-vaccine and pre-vaccine periods indicated that the mean age of carriage decreased significantly for serotype 3 (from 4.4 to 3.9 yrs) (Table 3, S1 Fig).

### Periodic cycles on the prevalence of carriage of non-vaccine serotypes

Serotypes 6C, 11A, 15A, 15B/C and NT were analyzed since these were the most frequently found in the dataset. The prevalence of serotypes 6C, 15B/C and NT was found to be associated with the vaccine period after adjusting for the child’s age, calendar year and day-care center (Table 2). Specifically, the high-vaccine period was associated with a significant increase in the prevalence of serotype 6C. Serotype 15B/C increased significantly from the pre-vaccine to the low-vaccine period. NT strains consistently increased overtime.

Analysis of the pre-vaccine and low-vaccine periods showed periodic cycles for serotypes 6C, 11A and NT with inter-epidemic periods of 5.9, 5.0 and 3.2 years, respectively (Fig 1). In the high-vaccine period, after 2007, the observed prevalence of serotype 6C no longer followed the cyclic pattern predicted by the model (Fig 1). For NT strains, in the high-vaccine period, a single peak was observed instead of the two peaks forecasted by the model.

Comparison of the mean age of carriage of non-vaccine serotypes between the high-vaccine and pre-vaccine periods showed that the mean age of carriage decreased significantly for serotypes 11A (from 4.1 to 3.4) and 15A (from 3.0 to 2.6). No differences in the mean age of carriage for the other non-vaccine serotypes were found (Table 3, S1 Fig).

## Discussion

To our best knowledge no study has looked at periodic cycles in pneumococcal carriage although those have been described in IPD [11–14]. The main finding of our study are: (i) serotypes frequently carried can exhibit periodic cycles and these can be modeled; (ii) these cycles can be perturbed directly or indirectly upon interventions such as introduction of a PCV; and (iii) shifts in the mean age of carriage of specific serotypes can occur as a consequence of these perturbations.

Regarding the first finding we observed that, before use of PCV7, periodic cycles (ranging from 3 to 6 years) were noticeable for PCV7 types 14, 19F and 23F, and non-PCV7 types 3, 6A, 6C, 11A, and NT. These observations can be better contextualized in light of previous studies. It has been shown that, upon colonization, serotype-specific immunity develops for serotypes 6A, 11A, 14, and 23F decreasing the probability of subsequent colonization by the same serotype [23–26]. In addition, a mathematical model aimed to simulate pneumococcal dynamics showed that when serotype-specific immunity occurs the prevalence of carriage has periodic peaks [26]. By contrast, serotype 19F is poorly immunogenic and less is known regarding serotypes 3, 6C and NT [23,25]. Since these latter serotypes were also associated with periodic cycles, other mechanisms may be contributing to it [24,26].

No periodic cycles were detected for other abundant serotypes: 15A, 19A and 6B. This might be due to several reasons that can hinder or prevent detection of oscillations in time: (i) long inter-epidemic periods (for example for 6B and 19A); (ii) low prevalence in carriage before introduction of PCV7 (observed for serogroup 15) [26]; and (iii) low serotype-specific immunity (described for serogroup 15) [25].

In IPD changes in the distribution of specific serotypes have been observed overtime but periodic cycles have not been systematically studied [11–14]. Still, a study from Denmark showed that serotypes 1, 2, 3 and 5 exhibited epidemic peaks in IPD every 2–3 years before 1960’s, and peaks every 7–10 years thereafter; whereas serogroups/serotypes 15, 19A, 22F and 33 showed a fairly stable proportion of cases overtime [14].

The observation of these cycles in IPD is a good indication that this should also occur in carriage since carriage precedes disease [11]. Direct comparison between periodic cycles in carriage and IPD is, nonetheless, difficult and currently such analyses are lacking in the available literature. It would be of interest to analyze systematic analyses on periodic cycles in IPD and carriage where robust temporal series are available.

The second main finding of our study was the observation that PCV can affect periodic cycles not only directly, but also indirectly. In agreement with other studies we observed that, upon use of PCV7, the prevalence of vaccine serotypes 6B, 14, and 23F decreased significantly. Still, two puzzling observations were made: a peak on the carriage prevalence of serotype 14 was noted in 2007, and an increase in 19F was noted during the high-vaccine period. This has not been observed in countries where PCV7 was universally introduced [27–29]. The non-universal use of PCV7 in Portugal is probably on the basis of these differences. In fact, models of pneumococcal dynamics predict that in settings where vaccine coverage is not large enough to interrupt circulation of vaccine serotypes their re-emergence may occur [26]. Why this was observed for only two PCV7 serotypes is not totally understood but should result from a combination of factors such as host and vaccine-mediated serotype-specific immunity, competition between serotypes, the selective pressure of antibiotic use, and serotype-associated patterns of antimicrobial resistance.

Of interest, our results suggested an indirect impact of PCV7 on periodic cycles of non-PCV7 serotypes 3, 6A, 6C, 11A, 19A and NT. This indirect impact was translated in three ways: (i) a higher amplitude in the PCV7 period (serotypes 3 and 11A), (ii) sustained increase in the prevalence of carriage (serotypes 6C, 19A and NT) and (iii) an increase in the inter-epidemic period (serotypes 3, 6A and NT).

The higher amplitude of the serotype 3 periodic cycle during the high-PCV7 period contrasts with what was recently reported in the case of IPD in Denmark where no such differences were observed in the PCV7 era [30]. The increase in the inter-epidemic period of serotype 6A in the high-vaccine period has been explained by the need of high anti-6B antibody concentrations for protection against this serotype [31].

Differences in the inter-epidemic period of specific serotypes between countries can result from several factors such as differences in: (i) the contact patterns among children [1]; (ii) the circulating serotypes (with implications on serotype specific- and non-specific immunity) [26] and (iii) abiotic factors, such as UV radiation [32]. In one study a lower UV radiation was a major predictor of IPD. The authors attributed this to the direct effects of UV on pathogen survival [32] impacting on transmission and on the inter-epidemic period in carriage.

The third main finding of this study was the observation of significant changes in child’s mean age of carriage of some serotypes. We observed an increase in the child’s mean age of carriage of PCV7-serotypes 6B, 19F, and 23F. An increased prevalence of PCV7 serotypes in older ages has also been observed in other settings where PCV7 has been introduced [27]. In addition, significant changes were also observed in the child’s mean age of carriage of serotypes 3, 6C, 11A and 15A, which became more frequent in ages previously associated with carriage of PCV7 serotypes. These shifts could be a consequence of an increase in the prevalence of those serotypes [33] and of the reduced circulation of competing PCV7-types that were previously occupying this niche [10,26]. The increase in carriage of serotypes 6C and 19A has also been observed in other countries after PCV7 implementation [27,29,34] but no details on shifts in the mean age of carriage were provided. Nonetheless, it is known that in IPD an age-related serotype distribution exists [35–37] as well as in carriage. In the absence of PCV7, it has been observed that PCV7 serotypes tend to peak in prevalence at 2 years of age and non-PCV7 serotypes at 4 years of age [38].

This study has some limitations inherent to the type of data that was used. Firstly, the time series was relatively short (15 years). This prevented use of standard methods of time series analysis and possibly did not enable detection of inter-epidemic periods for some serotypes. We tried to overcome these limitations by using multi-taper spectral analysis, which reduces estimation bias in the identification of peaks of short time series. Secondly, we used a convenience sample that may not be fully representative of the serotypes circulating in the population since only day-care centers were sampled. Ideally, a broader sample of the community would be desirable. Still, we consider that our sample was a good proxy of the serotypes circulating in the population for several reasons: (i) our studies included, at all time points, a diverse range of day-care centers which covered the different social strata that exist in Portugal; (ii) in Portugal the majority of young children attend day care centers and the trend is continuously increasing overtime (e.g., 75% in 2001 and 85% by 2010) [39]; (iii) several studies have shown that day-care centers are key major reservoirs of pneumococci contributing decisively to the community levels of pneumococcal carriage [1,40–42]. Finally, there may have been clustering of specific serotypes circulating in each day-care center. We dealt with this problem by correcting the variance in the regression models always including day-care center as a random variable. We also analyzed the presence of clusters every time we detected a significant peak in the time series.

In conclusion, ours findings provide evidence that carriage of pneumococcal serotypes can exhibit periodic cycles, which may imply that if PCV's coverage is not sufficiently high to interrupt transmission of PCV-serotypes, peaks of VTs with smaller amplitude and longer inter-epidemic period can occur. These peaks will include a higher proportion of older non-immunized children when compared with the pre-vaccine period and may imply that IPD patterns may change. Moreover, PCVs impact not only on PCV-serotypes but also affect the periodic cycles of non-PCV types. While the reasons underlying such effects are not fully understood, they likely reflect the results of complex intra-species interactions (between pneumococci of different serotypes) and of host-immunity. The results of this study should be taken into account when interpreting surveillance data on pneumococcal carriage.

## Supporting information

S1 Fig Serotype carriage by children’s age (in years) in the pre- and high-PCV7 periods. First panel: Overall age distribution between the pre-PCV7 (1996–2001) and the high-PCV7 period (2006–2010).
Other panels: Serotype-specific carriage as indicated.
Y-axis, age in years; X-axis, proportion of carriers.
(TIFF)

## References

[1] Huang SS, Finkelstein JA, Lipsitch M. Modeling community- and individual-level effects of child-care center attendance on pneumococcal carriage. Clin Infect Dis. 2005;40: 1215–22. doi: 10.1086/428580 15825020 10.1086/428580

[2] O’Brien KL, Wolfson LJ, Watt JP, Henkle E, Deloria-Knoll M, McCall N, et al Burden of disease caused by Streptococcus pneumoniae in children younger than 5 years: global estimates. Lancet. 2009;374: 893–902. doi: 10.1016/S0140-6736(09)61204-6 19748398 10.1016/S0140-6736(09)61204-6

[3] Feikin DR, Kagucia EW, Loo JD, Link-Gelles R, Puhan MA, Cherian T, et al Serotype-Specific Changes in Invasive Pneumococcal Disease after Pneumococcal Conjugate Vaccine Introduction: A Pooled Analysis of Multiple Surveillance Sites. PLoS Med. 2013;10. 10.1371/journal.pmed.1001517 PMC3782411 24086113

[4] Huang SS, Hinrichsen VL, Stevenson AE, Rifas-Shiman SL, Kleinman K, Pelton SI, et al Continued impact of pneumococcal conjugate vaccine on carriage in young children. Pediatrics. 2009;124: e1–11. doi: 10.1542/peds.2008-3099 19564254 10.1542/peds.2008-3099 PMC2782668

[5] Weinberger DM, Malley R, Lipsitch M. Serotype replacement in disease after pneumococcal vaccination. Lancet. 2011;378: 1962–73. doi: 10.1016/S0140-6736(10)62225-8 21492929 10.1016/S0140-6736(10)62225-8 PMC3256741

[6] Steens A, Bergsaker MAR, Aaberge IS, Rønning K, Vestrheim DF. Prompt effect of replacing the 7-valent pneumococcal conjugate vaccine with the 13-valent vaccine on the epidemiology of invasive pneumococcal disease in Norway. Vaccine. 2013;31: 6232–8. doi: 10.1016/j.vaccine.2013.10.032 24176490 10.1016/j.vaccine.2013.10.032

[7] Chang Q, Stevenson AE, Croucher NJ, Lee GM, Pelton SI, Lipsitch M, et al Stability of the pneumococcal population structure in Massachusetts as PCV13 was introduced. BMC Infect Dis. 2015;15: 68 doi: 10.1186/s12879-015-0797-z 25887323 10.1186/s12879-015-0797-z PMC4336693

[8] Domenech M, Damián D, Ardanuy C, Liñares J, Fenoll A, García E. Emerging, Non-PCV13 Serotypes 11A and 35B of Streptococcus pneumoniae Show High Potential for Biofilm Formation In Vitro. PLoS One. 2015;10: e0125636 doi: 10.1371/journal.pone.0125636 25927917 10.1371/journal.pone.0125636 PMC4415931

[9] Cohen R, Levy C, Bingen E, Koskas M, Nave I, Varon E. Impact of 13-valent pneumococcal conjugate vaccine on pneumococcal nasopharyngeal carriage in children with acute otitis media. Pediatr Infect Dis J. 2012;31: 297–301. doi: 10.1097/INF.0b013e318247ef84 22330166 10.1097/INF.0b013e318247ef84

[10] Lipsitch M, Dykes JK, Johnson SE, Ades EW, King J, Briles DE, et al Competition among Streptococcus pneumoniae for intranasal colonization in a mouse model. Vaccine. 2000;18: 2895–901. 10812233 10.1016/s0264-410x(00)00046-3

[11] Feikin DR, Klugman KP. Historical changes in pneumococcal serogroup distribution: implications for the era of pneumococcal conjugate vaccines. Clin Infect Dis. 2002;35: 547–55. doi: 10.1086/341896 12173128 10.1086/341896

[12] Fenoll A, Granizo JJ, Aguilar L, Giménez MJ, Aragoneses-Fenoll L, Hanquet G, et al Temporal trends of invasive Streptococcus pneumoniae serotypes and antimicrobial resistance patterns in Spain from 1979 to 2007. J Clin Microbiol. 2009;47: 1012–20. doi: 10.1128/JCM.01454-08 19225097 10.1128/JCM.01454-08 PMC2668361

[13] Jefferies JM, Smith AJ, Edwards GFS, McMenamin J, Mitchell TJ, Clarke SC. Temporal analysis of invasive pneumococcal clones from Scotland illustrates fluctuations in diversity of serotype and genotype in the absence of pneumococcal conjugate vaccine. J Clin Microbiol. 2010;48: 87–96. doi: 10.1128/JCM.01485-09 19923488 10.1128/JCM.01485-09 PMC2812259

[14] Harboe ZB, Benfield TL, Valentiner-Branth P, Hjuler T, Lambertsen L, Kaltoft M, et al Temporal trends in invasive pneumococcal disease and pneumococcal serotypes over 7 decades. Clin Infect Dis. 2010;50: 329–37. doi: 10.1086/649872 20047478 10.1086/649872

[15] Comissão de Vacinas da Sociedade de Infeciologia Pediátrica (SIP) e Sociedade Portuguesa de Pediatria (SPP). Recomendações sobre vacinas: actualização 2014. 2014.

[16] Nunes S, Félix S, Valente C, Simões AS, Tavares DA, Almeida ST, et al The impact of private use of PCV7 in 2009 and 2010 on serotypes and antimicrobial resistance of Streptococcus pneumoniae carried by young children in Portugal: Comparison with data obtained since 1996 generating a 15-year study prior to PCV13 introduction. Vaccine. 2016;34: 1648–1656. doi: 10.1016/j.vaccine.2016.02.045 26920470 10.1016/j.vaccine.2016.02.045

[17] Pai R, Gertz RE, Beall B. Sequential Multiplex PCR Approach for Determining Capsular Serotypes of Streptococcus pneumoniae Isolates. J Clin Microbiol. 2006;44: 124–131. doi: 10.1128/JCM.44.1.124-131.2006 16390959 10.1128/JCM.44.1.124-131.2006 PMC1351965

[18] CDC C for DC and P. Streptococcus Lab | StrepLab | PCR Deduction of Pneumococcal Serotypes | CDC [Internet]. 2014 [cited 8 Mar 2017]. Available: https://www.cdc.gov/streplab/pcr.html

[19] Simões AS, Valente C, de Lencastre H, Sá-Leão R. Rapid identification of noncapsulated Streptococcus pneumoniae in nasopharyngeal samples allowing detection of co-colonization and reevaluation of prevalence. Diagn Microbiol Infect Dis. 2011;71: 208–216. doi: 10.1016/j.diagmicrobio.2011.07.009 21907526 10.1016/j.diagmicrobio.2011.07.009

[20] Wood SN. Generalized additive models: an introduction with R [Internet]. Boca Raton, FL: Chapman & Hall/CRC; 2006 Available: http://www.loc.gov/catdir/enhancements/fy0702/2006040209-d.html

[21] Babadi B, Brown EN. A review of multitaper spectral analysis. IEEE Trans Biomed Eng. 2014;61: 1555–64. doi: 10.1109/TBME.2014.2311996 24759284 10.1109/TBME.2014.2311996

[22] R Core Team. R: A Language and Environment for Statistical Computing. Vienna, Austria.: R Foundation for Statistical Computing; 2014.

[23] Soininen A, Pursiainen H, Kilpi T, Käyhty H. Natural development of antibodies to pneumococcal capsular polysaccharides depends on the serotype: association with pneumococcal carriage and acute otitis media in young children. J Infect Dis. 2001;184: 569–76. doi: 10.1086/322794 11494163 10.1086/322794

[24] Lipsitch M, Whitney CG, Zell E, Kaijalainen T, Dagan R, Malley R. Are anticapsular antibodies the primary mechanism of protection against invasive pneumococcal disease? PLoS Med. 2005;2: e15 doi: 10.1371/journal.pmed.0020015 15696204 10.1371/journal.pmed.0020015 PMC545206

[25] Weinberger DM, Dagan R, Givon-Lavi N, Regev-Yochay G, Malley R, Lipsitch M. Epidemiologic evidence for serotype-specific acquired immunity to pneumococcal carriage. J Infect Dis. 2008;197: 1511–8. doi: 10.1086/587941 18471062 10.1086/587941

[26] Flasche S, Edmunds WJ, Miller E, Goldblatt D, Robertson C, Choi YH. The impact of specific and non-specific immunity on the ecology of Streptococcus pneumoniae and the implications for vaccination. Proc Biol Sci. 2013;280: 20131939 doi: 10.1098/rspb.2013.1939 24089337 10.1098/rspb.2013.1939 PMC3790488

[27] Richter SS, Heilmann KP, Dohrn CL, Riahi F, Diekema DJ, Doern GV. Pneumococcal Serotypes before and after Introduction of Conjugate Vaccines, United States, 1999–20111. Emerg Infect Dis. 2013;19: 1074–1083. doi: 10.3201/eid1907.121830 23763847 10.3201/eid1907.121830 PMC3713983

[28] Gladstone RA, Jefferies JM, Tocheva AS, Beard KR, Garley D, Chong WW, et al Five winters of pneumococcal serotype replacement in UK carriage following PCV introduction. Vaccine. 2015;33: 2015–2021. doi: 10.1016/j.vaccine.2015.03.012 25776920 10.1016/j.vaccine.2015.03.012 PMC4392391

[29] Torné AN, Dias JG, Quinten C, Hruba F, Busana MC, Lopalco PL, et al European enhanced surveillance of invasive pneumococcal disease in 2010: Data from 26 European countries in the post-heptavalent conjugate vaccine era. Vaccine. 2014;32: 3644–3650. doi: 10.1016/j.vaccine.2014.04.066 24795228 10.1016/j.vaccine.2014.04.066

[30] Harboe ZB, Dalby T, Weinberger DM, Benfield T, Mølbak K, Slotved HC, et al Impact of 13-valent pneumococcal conjugate vaccination in invasive pneumococcal disease incidence and mortality. Clin Infect Dis. 2014;59: 1066–1073. doi: 10.1093/cid/ciu524 25034421 10.1093/cid/ciu524

[31] Dagan R, Givon-Lavi N, Porat N, Greenberg D. The effect of an alternative reduced-dose infant schedule and a second year catch-up schedule with 7-valent pneumococcal conjugate vaccine on pneumococcal carriage: A randomized controlled trial. Vaccine. 2012;30: 5132–5140. doi: 10.1016/j.vaccine.2012.05.059 22683519 10.1016/j.vaccine.2012.05.059

[32] White ANJ, Ng V, Spain CV, Johnson CC, Kinlin LM, Fisman DN. Let the sun shine in: effects of ultraviolet radiation on invasive pneumococcal disease risk in Philadelphia, Pennsylvania. BMC Infect Dis. 2009;9: 196 doi: 10.1186/1471-2334-9-196 19961583 10.1186/1471-2334-9-196 PMC2797517

[33] Bogaert D, Engelen MN, Timmers-Reker AJ, Elzenaar KP, Peerbooms PG, Coutinho RA, et al Pneumococcal carriage in children in The Netherlands: a molecular epidemiological study. J Clin Microbiol. 2001;39: 3316–20. doi: 10.1128/JCM.39.9.3316-3320.2001 11526169 10.1128/JCM.39.9.3316-3320.2001 PMC88337

[34] Tocheva AS, Jefferies JMC, Christodoulides M, Faust SN, Clarke SC. Increase in Serotype 6C Pneumococcal Carriage, United Kingdom. Emerg Infect Dis. 2010;16: 154–155. doi: 10.3201/eid1601.090650 20031068 10.3201/eid1601.090650 PMC2874357

[35] Usonis V, Stacevičienė I, Petraitienė S, Vaičiūnienė D, Alasevičius T, Kirslienė J. Streptococcus pneumoniae nasopharyngeal colonisation in children aged under six years with acute respiratory tract infection in Lithuania, February 2012 to March 2013. Euro Surveill. 2015;20: 34–41. 25860394 10.2807/1560-7917.es2015.20.13.21079

[36] Imöhl M, Reinert RR, Ocklenburg C, van der Linden M. Association of Serotypes of Streptococcus pneumoniae with Age in Invasive Pneumococcal Disease. J Clin Microbiol. 2010;48: 1291–1296. doi: 10.1128/JCM.01937-09 20107087 10.1128/JCM.01937-09 PMC2849605

[37] Lagos R, Muñoz A, San Martin O, Maldonado A, Hormazabal JC, Blackwelder WC, et al Age- and serotype-specific pediatric invasive pneumococcal disease: insights from systematic surveillance in Santiago, Chile, 1994–2007. J Infect Dis. 2008;198: 1809–1817. doi: 10.1086/593334 18959497 10.1086/593334

[38] Bogaert D, van Belkum A, Sluijter M, Luijendijk A, de Groot R, Rümke HC, et al Colonisation by Streptococcus pneumoniae and Staphylococcus aureus in healthy children. Lancet Lond Engl. 2004;363: 1871–1872. 10.1016/S0140-6736(04)16357-5 15183627

[39] dos Santos FFM. Matriculados: Pré-escolar por subsistema de ensino. PORDATA [Internet]. 2016 [cited 16 Apr 2016]. Available: http://www.pordata.pt/Home

[40] Pessoa D, Hoti F, Syrjänen R, Sá-Leão R, Kaijalainen T, Gomes MGM, et al Comparative analysis of Streptococcus pneumoniae transmission in Portuguese and Finnish day-care centres. BMC Infect Dis. 2013;13: 180 doi: 10.1186/1471-2334-13-180 23597389 10.1186/1471-2334-13-180 PMC3652738

[41] Givon-Lavi N, Fraser D, Dagan R. Vaccination of day-care center attendees reduces carriage of Streptococcus pneumoniae among their younger siblings. Pediatr Infect Dis J. 2003;22: 524–532. doi: 10.1097/01.inf.0000069760.65826.f2 12799509 10.1097/01.inf.0000069760.65826.f2

[42] Sá-Leão R, Tomasz A, Sanches IS, Nunes S, Alves CR, Avô AB, et al Genetic Diversity and Clonal Patterns among Antibiotic-Susceptible and -Resistant Streptococcus pneumoniae Colonizing Children: Day Care Centers as Autonomous Epidemiological Units. J Clin Microbiol. 2000;38: 4137–4144. 11060081 10.1128/jcm.38.11.4137-4144.2000 PMC87554

