# Supplementary figures and images for: Periodic cycles of pneumococcal serotypes carried by children before and after 7-valent pneumococcal conjugate vaccine
Source: PLoS One. 2017 Apr 28;12(4):e0176723. doi: 10.1371/journal.pone.0176723 (PMC5409052; doi:10.1371/journal.pone.0176723)

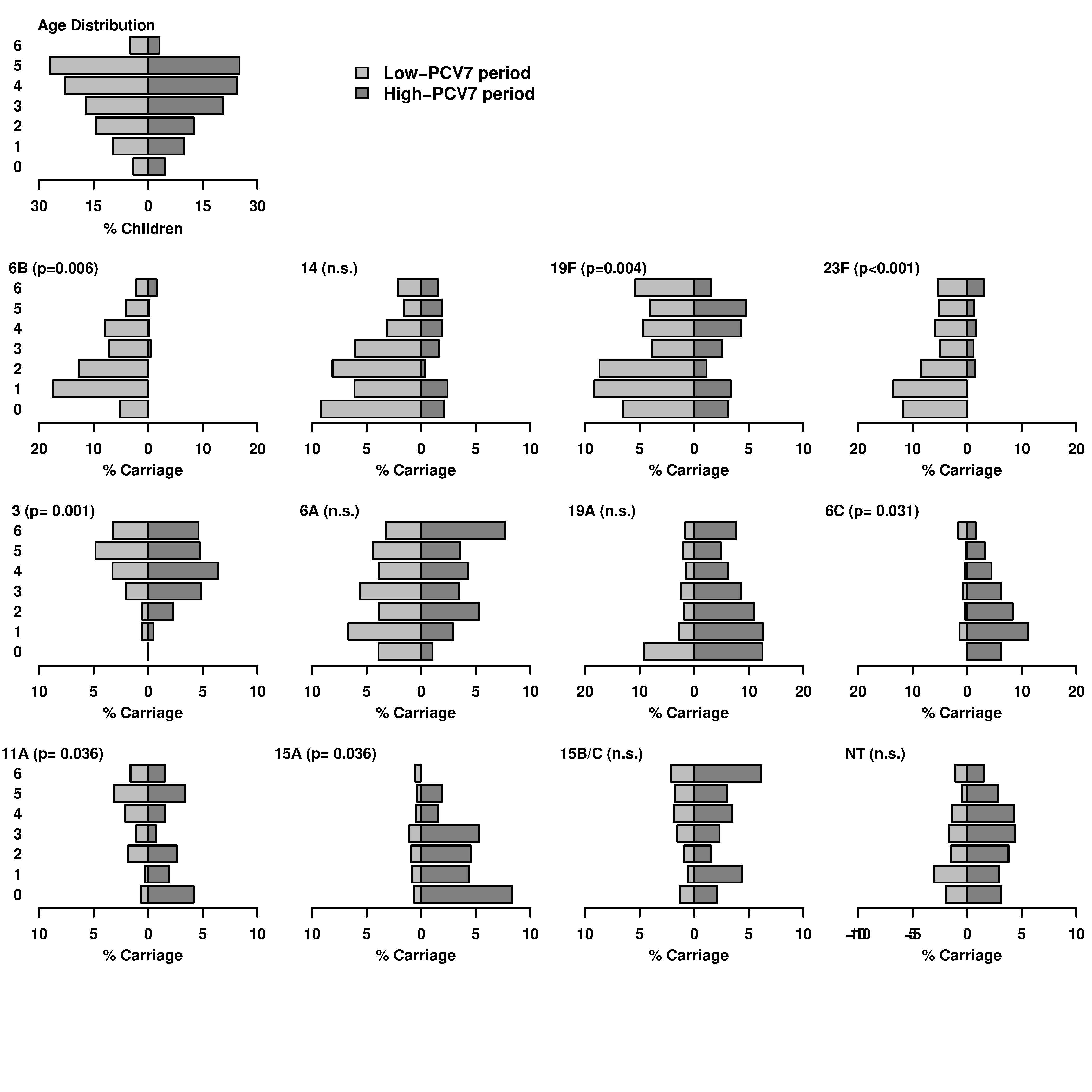

Supplement: S1 Fig — First panel: Overall age distribution between the pre-PCV7 (1996–2001) and the high-PCV7 period (2006–2010). Other panels: Serotype-specific carriage as indicated. Y-axis, age in years; X-axis, proportion of carriers. (TIFF) [file pone.0176723.s001.tiff]
